# Observation of Cellodextrin Accumulation Resulted from Non-Conventional Secretion of Intracellular β-Glucosidase by Engineered *Saccharomyces cerevisiae* Fermenting Cellobiose

**DOI:** 10.4014/jmb.2105.05018

**Published:** 2021-07-06

**Authors:** Won-Heong Lee, Yong-Su Jin

**Affiliations:** 1 Department of Food Science and Human Nutrition, and Carl R. Woese Institute for Genomic Biology, University of Illinois at Urbana-Champaign, Urbana, IL 61801, USA; 2 Department of Bioenergy Science and Technology, and Department of Integrative Food, Bioscience and Biotechnology, Chonnam National University, Gwangju 61186, Republic of Korea

**Keywords:** Cellulosic ethanol, engineered *Saccharomyces cerevisiae*, intracellular β-glucosidase, transglycosylation, non-conventional protein secretion

## Abstract

Although engineered *Saccharomyces cerevisiae* fermenting cellobiose is useful for the production of biofuels from cellulosic biomass, cellodextrin accumulation is one of the main problems reducing ethanol yield and productivity in cellobiose fermentation with *S. cerevisiae* expressing cellodextrin transporter (CDT) and intracellular β-glucosidase (GH1-1). In this study, we investigated the reason for the cellodextrin accumulation and how to alleviate its formation during cellobiose fermentation using engineered *S. cerevisiae* fermenting cellobiose. From the series of cellobiose fermentation using *S. cerevisiae* expressing only GH1-1 under several culture conditions, it was discovered that small amounts of GH1-1 were secreted and cellodextrin was generated through trans-glycosylation activity of the secreted GH1-1. As GH1-1 does not have a secretion signal peptide, non-conventional protein secretion might facilitate the secretion of GH1-1. In cellobiose fermentations with *S. cerevisiae* expressing only GH1-1, knockout of *TLG2* gene involved in non-conventional protein secretion pathway significantly delayed cellodextrin formation by reducing the secretion of GH1-1 by more than 50%. However, in cellobiose fermentations with *S. cerevisiae* expressing both GH1-1 and CDT-1, *TLG2* knockout did not show a significant effect on cellodextrin formation, although secretion of GH1-1 was reduced by more than 40%. These results suggest that the development of new intracellular β-glucosidase, not influenced by non-conventional protein secretion, is required for better cellobiose fermentation performances of engineered *S. cerevisiae* fermenting cellobiose.

## Introduction

Production of biofuels and chemicals from renewable biomass has received considerable attention as an alternative and sustainable way compared with the production of petroleum-based fuels and chemicals [1-3]. In particular, baker’s yeast, *Saccharomyces cerevisiae*, has been employed as a microbial host to produce biofuels and chemicals from cellulosic biomass as this yeast has been utilized in ethanol production from starch-based biomass [1, 4, 5]. Because native *S. cerevisiae* cannot metabolize xylose, one of the abundant sugars (glucose and xylose) from enzymatic hydrolysis of cellulosic biomass, it has been engineered to metabolize xylose for efficient production of cellulosic ethanol [4-6]. Nevertheless, xylose metabolism by engineered *S. cerevisiae* is still limited by catabolite repression (glucose repression) in the presence of glucose [7-10], which has demanded new strategies for the efficient utilization of xylose by engineered *S. cerevisiae* without glucose repression.

Introduction of intracellular cellobiose assimilating pathway, composed of cellodextrin transporter (CDT-1 or CDT-2 from cellulolytic fungi, *Neurospora crassa*) and intracellular β-glucosidase (GH1-1 from *N. crassa*), into *S. cerevisiae* has been verified to be an effective approach for the production of cellulosic ethanol because it enables *S. cerevisiae* to ferment cellobiose and xylose simultaneously without glucose repression [11-13]. Furthermore, engineered *S. cerevisiae* fermenting cellobiose can enhance ethanol yield and productivity by co-fermentation of cellobiose and xylose compared with sequential fermentation of glucose and xylose [12, 13]. In addition, cellobiose-fermenting *S. cerevisiae* reduces enzyme costs and enhances ethanol productivity in simultaneous saccharification and fermentation (SSF) of cellulose [14, 15]. However, engineered *S. cerevisiae* that utilizes intracellular cellobiose poses several problems in cellobiose fermentation despite of the benefits mentioned above. These problems include the accumulation of cellodextrin (*e.g.*, cellotriose and cellotetraose) during cellobiose fermentation and slower cellobiose fermentation than glucose fermentation [12, 16-18]. In particular, cellodextrin accumulation increased when the cellobiose consumption rate by engineered *S. cerevisiae* was increased, which caused retardation of ethanol production during cellobiose fermentation [16-18].

Considering that cellodextrin is formed by trans-glycosylation of β-glucosidase when the concentration of cellobiose is extremely high [19-21], it would be conceivable that cellodextrin accumulation by engineered *S. cerevisiae* fermenting cellobiose might be caused by trans-glycosylation activities of intracellular and/or extracellular β-glucosidase. As such, we hypothesized that small amounts of GH1-1, even though GH1-1 lacks secretion signal-peptides, might be secreted (or leaked due to cell lysis) from the engineered *S. cerevisiae* cells by an unknown mechanism and secretion of GH1-1 might contribute to the accumulation of cellodextrin during cellobiose fermentation.

This study was undertaken to investigate the possibility for secretion of intracellular β-glucosidase by engineered *S. cerevisiae* utilizing intracellular cellobiose. To this end, we examined various culture conditions stimulating the secretion of GH1-1 by *S. cerevisiae* strain expressing only GH1-1 (without cellodextrin transporter). Moreover, we investigated the knockout effects of *TLG2*, which is involved in non-conventional protein secretion in *S. cerevisiae*, on the secretion of GH1-1 and cellodextrin accumulation by engineered *S. cerevisiae* strain fermenting cellobiose.

## Materials and Methods

### Strains and Plasmids

*S. cerevisiae* BY4742 (*MATα, his3, leu2, lys2, ura3*) and *TLG2*-knockout *S. cerevisiae* BY4742 (Δ*TLG2*) (Open Biosystems, USA) were used as the host strains for expressing an intracellular β-glucosidase (GH1-1) with (or without) a cellodextrin transporter (CDT-1) from *N. crassa*. Plasmids pRS425-gh1-1 and pRS426-cdt1 were previously constructed for overexpression of intracellular β-glucosidase and cellodextrin transporter, respectively [11]. All strains and plasmids used in this study are listed in Table 1.

### Culture Conditions

Synthetic complete (SC) medium (6.7 g/l yeast nitrogen base without amino acids and 0.625 g/l complete supplement mixture without histidine, leucine and uracil) with appropriate nucleotide, amino acids and 20 g/l glucose (dextrose) was used for seed cultivation. A single colony of each *S. cerevisiae* BY4742 strains from SCD agar plates was picked and inoculated into 5 ml of SCD medium (20 g/l glucose, pH 6.5). Seed cultivation was performed in 10-mL test tube at 30°C and 250 rpm.

For pre-cultivation of the engineered yeast strains, SCD medium (40 g/l glucose, pH 6.5) was used. After 48 h of seed cultivation, cells were harvested and inoculated into 25 ml of SCD medium. Pre-cultivation was performed in 125-mL flask at 30°C and 250 rpm. SC media at pH 6.5 (or pH 5.5) containing 20 g/l cellobiose or YP media (10 g/l yeast extract and 20 g/l Bacto-peptone, pH 6.5 or 5.5) containing 20 g/l cellobiose (or 80 g/l cellobiose) were used for cellobiose fermentation of *S. cerevisiae* BY4742 strains expressing GH1-1 with or without CDT-1 [BY-B, BY-B (Δ*TLG2*), BY-BT, and BY-BT (Δ*TLG2*) strains]. YPD medium (20 g/l glucose, pH 6.5) was also used to compare glucose fermentations of BY-B and BY-B (Δ*TLG2*) strains with cellobiose fermentations. After 24 h of pre-cultivation, yeast cells were harvested. After washing three times with sterilized water, the cells were inoculated into 50 ml of fermentation medium at initial optical density (OD)_600_ of ~0.5 (corresponds to 0.173 g/l of dry cell mass). Cultivation was performed in 250-ml flask at 30°C and 80 rpm (micro-aerobic).

To determine the secretion of GH1-1 by the yeast strains, culture broth at the end of the fermentation was harvested and used to examine extracellular β-glucosidase activity.

### Analytical Methods

Cell concentration was monitored by measuring OD at 600 nm using a UV-visible spectrophotometer (Biomate5, Thermo, USA). Glucose, cellobiose, cellodextrin and ethanol concentrations were determined by high performance liquid chromatography (HPLC; Agilent Technologies 1200 Series, Agilent, USA) equipped with a refractive index (RI) detector using a Rezex ROA-Organic Acid H^+^ (8%) column (Phenomenex Inc., USA). The column was eluted with 0.005 N of H_2_SO_4_ at a flow rate of 0.6 ml/min at 50°C.

Extracellular β-glucosidase activity was measured according to the methods reported previously [20-22]. The culture broth was harvested and centrifuged at 15,000 ×*g* for 20 min at 4°C. After filtration of the supernatant with a 0.22-μm spin filter, the filtrate (500 μl) was mixed with the same volume of 50 mM sodium citrate buffer (pH 4.8). After addition of 6.7 mM *p*-nitrophenyl-β-D-glucopyranoside (*p*-NPG), release of *p*-nitrophenol (*p*-NP) was monitored by a spectrophotometer at 400 nm. One unit (U) of β-glucosidase was defined as the amount of enzyme that catalyzes the release of 1 μmol of *p*-NP per min at 30°C. Volumetric extracellular β-glucosidase activity was expressed as units of β-glucosidase per liter. Specific extracellular β-glucosidase activity was expressed as units of β-glucosidase per gram of dry cell.

## Results and Discussion

### Confirmation of GH1-1 Secretion from Engineered *S. cerevisiae* Expressing GH1-1

Native *S. cerevisiae* strains, including BY4742, are unable to utilize cellobiose as they lack genes involved in the transport or degradation of cellobiose. Because there is no signal sequence for secretion of intracellular β-glucosidase (GH1-1) on the expression cassette of GH1-1 (pRS425-gh1-1) [11], GH1-1 must be located inside the cell when it is expressed in *S. cerevisiae*. Therefore, *S. cerevisiae* expressing only GH1-1 without cellobiose transporter (CDT-1) cannot metabolize cellobiose and there is no possibility of cellobiose degradation and cellodextrin formation by the GH1-1 expressing *S. cerevisiae* because intracellular β-glucosidase cannot react with extracellular cellobiose.

**Fig. 1:**
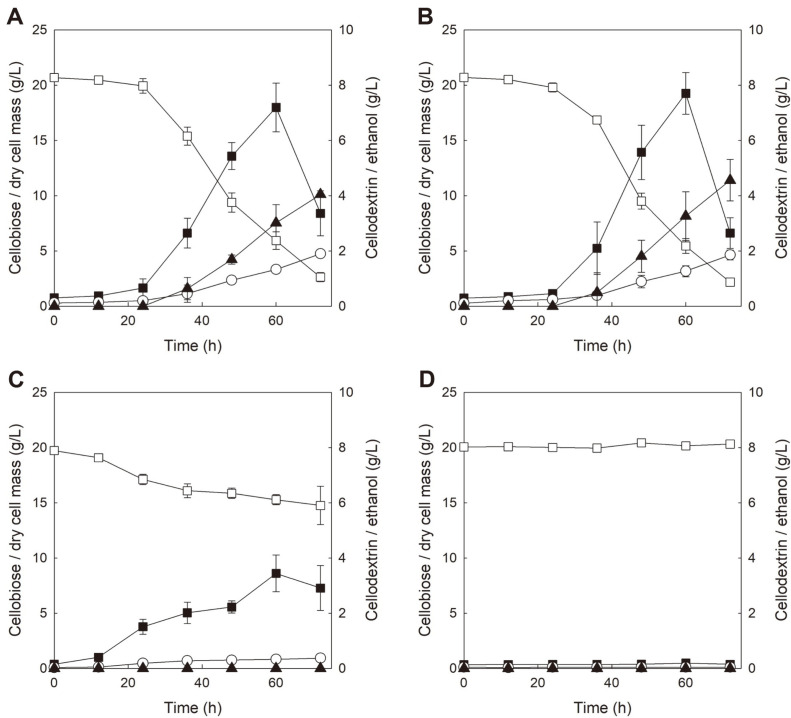
Comparison of cellobiose fermentation profiles of *S. cerevisiae* expressing GH1-1 (BY-B) under different culture conditions. Complex (YP) medium with 20 g/l cellobiose at pH 6.5 (**A**), YP medium with 20 g/l cellobiose at pH 5.5 (**B**), minimal (SC) medium with 20 g/l cellobiose at pH 6.5 (**C**), and SC medium with 20 g/l cellobiose at pH 5.5 (**D**). Symbols: cellobiose (□), dry cell mass (○), cellodextrin (■), and ethanol (▲). All values of fermentations are mean values from two independent fermentation experiments, and error bars represent standard deviations.

To verify whether *S. cerevisiae* expressing only GH1-1 could consume cellobiose and whether cellodextrin formation could occur or not, the growth of the *S. cerevisiae* BY-B strain (BY4742 strain expressing GH1-1 only) on cellobiose was tested in a complex media (YP containing 20 g/l cellobiose) at pH 6.5. Although BY-B could not transport cellobiose into the cell at all, cell growth, ethanol production, and accumulation of cellodextrin along with cellobiose consumption were observed in cellobiose fermentation with BY-B. As shown in Fig. 1A, 18.1 g/l of cellobiose was consumed and 4.0 g/l of ethanol was produced during cellobiose fermentation. Cellodextrin was accumulated up to 7.2 g/l at 60 h and decreased to 3.4 g/l at 72 h of fermentation. Considering that it is impossible for most sugars to pass through the yeast membrane by simple diffusion [23], we reasoned that formation and degradation of cellodextrin as well as degradation of cellobiose might occur at the outside of BY-B cells. Probably, BY-B may secrete a small amount of GH1-1 and the secreted GH1-1 might be involved in the degradation of cellobiose to glucose along with accumulation and degradation of cellodextrin. To validate whether small amounts of GH1-1 were secreted from the cell or not, the extracellular β-glucosidase activity of BY-B was measured using the culture broth at the end of the fermentation. As shown in Table 2, BY-B grown in YPC20 (pH 6.5) exhibited considerable level of extracellular β-glucosidase activity (14.9 U/L and 3.1 U/g cell). Considering that the average level of specific intracellular β-glucosidase activity of *S. cerevisiae* expressing GH1-1 grown in YPC was around 180 U/g cell (data not shown), it was estimated that approximately 2% of GH1-1 expressed in the cell was secreted outside the cell. This observation is in good accordance to a previous report describing that frequency of trans-glycosylation reaction (cellodextrin formation) is dominant in the cellobiose hydrolysis reaction when cellobiose concentration is significantly higher than β-glucosidase concentration [19, 21]. Results from cellobiose fermentation and extracellular β-glucosidase activity assay of BY-B clearly indicates that secretion of GH1-1 can contribute to extracellular cellobiose degradation and cellodextrin formation.

The growth of BY-B in YP containing 20 g/l cellobiose under lower pH conditions was also tested to check if cellobiose consumption and cellodextrin formation by BY-B were affected by the change of external pH. Fig. 1B shows the cellobiose fermentation profile of BY-B in YPC20 at pH 5.5. It is interesting that almost similar profiles of cell growth, ethanol production, and cellodextrin accumulation were observed compared with those of cellobiose fermentation at pH 6.5. It was observed that 18.5 g/l of cellobiose was consumed and 4.6 g/l of ethanol was produced by BY-B from 20 g/l of cellobiose. Cellodextrin was accumulated to 7.7 g/l until 60 h and then rapidly decreased to 2.6 g/l. The transition from cellodextrin accumulation to cellodextrin degradation occurred when the cell concentrations were higher than 3 g/l and the concentrations of cellobiose were lower than those of cellodextrin as shown in Figs. 1A and 1B. These results suggest that until 60 h of fermentation, sufficient amounts of β-glucosidase for degradation of cellodextrin (and cellobiose) might not be secreted by the engineered yeast. As trans-glycosylation and hydrolysis reaction are controlled by the concentrations of β-glucosidase and cellobiose, the transition from trans-glycosylation to hydrolysis reaction might have occurred around 60 h of the fermentation. BY-B grown in YPC20 (pH 5.5) also exhibited almost the same level of extracellular β-glucosidase activity (14.5 U/L and 3.1 U/g cell) compared with that of cells grown in YPC20 at pH 6.5. Probably, activated protein synthesis in cells cultivated in a complex medium may lead to leakage of GH1-1 because YP medium provides many nutrients for protein synthesis, such as peptides, amino acids and nucleotides. As such, we reasoned that secretion of GH1-1 might be prevented (or reduced) if a minimal medium was used for cellobiose fermentation with BY-B. In addition, it could not be excluded that the putative cellobiose transporter in *S. cerevisiae* may transport cellobiose into the cell and then intracellular cellobiose was hydrolyzed to glucose or converted to cellodextrin by GH1-1.

To verify whether similar patterns of cellobiose utilization could be observed when the type of medium and external pH conditions were changed, cellobiose fermentation of BY-B was tested in SC containing 20 g/l of cellobiose at different pH conditions (6.5 and 5.5). Fig. 1C shows the cellobiose fermentation profile of BY-B in SCC20 at pH 6.5. As expected, reduced cellobiose consumption was observed in the SCC medium. Although only 5.1 g/l of cellobiose was consumed and ethanol was not produced during the fermentation, cellodextrin was accumulated at 3.4 g/l, which was around 70% conversion of the consumed cellobiose to cellodextrin. In additional cellobiose fermentation of BY-B which was extended until 144 hours in SCC20 at pH 6.5, after cellodextrin was accumulated up to 3.4 g/l at 60 h, cellobiose concentration slowly increased to 3.7 g/l at 144 h of fermentation (data not shown). Compared with the results from cellobiose fermentation in YPC medium, less amount of GH1-1 seemed to be secreted from the cell due to reduced protein synthesis in SCC medium, which could explain the much lower cell concentration, no ethanol production and increased conversion ratio of cellobiose to cellodextrin. Fig. 1D shows the cellobiose fermentation profile of BY-B in SCC20 at pH 5.5. Interestingly, the BY-B strain exhibited neither cellobiose consumption nor cell growth. Moreover, cellodextrin accumulation was not observed. These results suggest that *S. cerevisiae* might not have any putative cellobiose transporter because the putative transporter (if it exists) might function at pH 5.5 (optimum pH for *S. cerevisiae* growth) as well, even-though yeast cells cultured at pH 5.5 can show different cellobiose transport efficiency compared with those cultured at pH 6.5. These results also suggest that secretion of GH1-1 may be affected by growth conditions, such as external pH and supply of nutrients. To confirm that the level of GH1-1 secretion was affected by cell growth conditions, extracellular β-glucosidase activities of BY-B grown in SCC were measured. As shown in Table 2, BY-B cells grown in SCC at pH 6.5 showed much lower level of extracellular β-glucosidase activity (0.6 U/L) than cells grown in YPC. In particular, BY-B cells grown at pH 5.5 showed no extracellular β-glucosidase activity. These results indicate that secretion of GH1-1 might not occur in a minimal medium with lower pH conditions. In fact, secretion of GH1-1 from BY-B seemed to trigger cell growth, ethanol production and cellodextrin accumulation through extracellular cellobiose degradation and trans-glycosylation. The results from cellobiose fermentations of BY-B under different growth conditions are summarized in Table 3.

From previous studies on cellobiose fermentation with engineered *S. cerevisiae* strains expressing GH1-1 and cellodextrin transporters (CDT-1 or CDT-2 from *N. crassa*) [12, 16-18], it was hypothesized that cellodextrin accumulation may be primarily due to intracellular trans-glycosylation by GH1-1 through a series of steps, such as transportation of cellobiose, intracellular formation of cellodextrin, and export of cellodextrin by transporter (or diffusion). However, considering that small amounts of GH1-1 was secreted by BY-B in this study, it is also proposed that cellodextrin accumulation during cellobiose fermentation with engineered *S. cerevisiae* expressing GH1-1 and CDT may also be caused by secretion (or leakage) of GH1-1.

### Effect of *TLG2* Knockout on Cellobiose Fermentation with Engineered *S. cerevisiae*

As mentioned above, expression cassette for GH1-1 (pRS425-gh1-1) did not have any signal sequence for secretion, suggesting that GH1-1 may be secreted through a non-conventional protein secretion pathway [24-26], not a normal protein secretion pathway (endoplasmic reticulum- and Golgi-dependent protein secretion with N-terminal signal peptide) [26, 27] in *S. cerevisiae*. It has been reported that various proteins lacking signal peptide could be transported to the cell surface for their extracellular functions in yeast, such as *S. cerevisiae* and *Candida albicans* [25, 26]. For example, signal peptide-less proteins in cytoplasm, such as glycolytic enzymes, chaperones and translation factors, have been frequently observed at the surface of yeast cells [24-26]. These studies proposed several potential mechanisms for non-conventional protein secretion; however, most mechanisms have not yet been clearly characterized [24-26]. Tlg2p is an endosomal specific t-SNARE protein that participates in fusion of endosome-derived vesicles, endocytosis, and exocytosis [24, 25, 28]. Moreover, Tlg2p is involved in non-conventional secretion of several metabolic enzymes in the cytoplasm [24, 25, 28, 29]. Previous studies have revealed that *TLG2* knockout significantly reduces non-conventional secretion of Acb1p (acyl-CoA-binding protein in fatty acid biosynthesis) and Eno1p (enolase in glycolysis) in *S. cerevisiae* [28, 29].

**Fig. 2:**
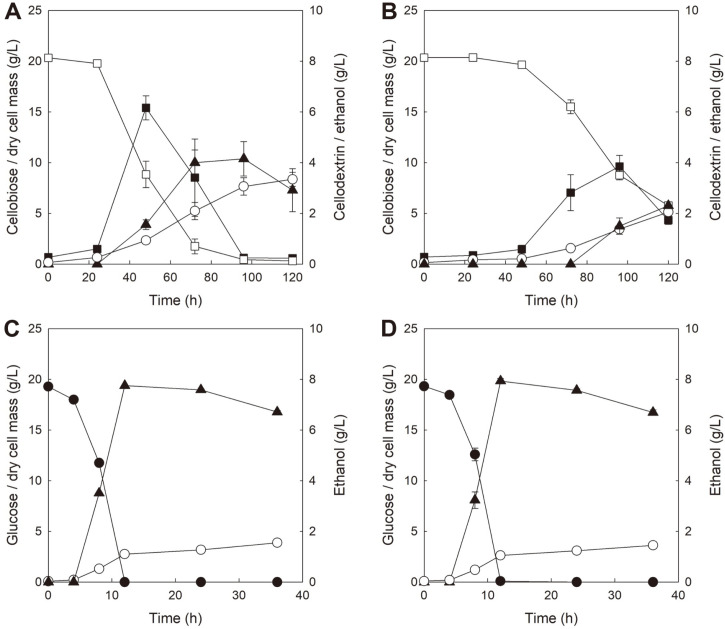
Comparison of fermentation profiles of *S. cerevisiae* strains expressing GH1-1 [BY-B and BY-B (Δ*TLG2*)] under different culture conditions. BY-B in YP medium with 20 g/l cellobiose at pH 6.5 (**A**), BY-B (Δ*TLG2*) in YP medium with 20 g/l cellobiose at pH 6.5 (**B**), BY-B in YP medium with 20 g/l glucose at pH 6.5 (**C**), and BY-B (Δ*TLG2*) in YP medium with 20 g/l glucose at pH 6.5 (**D**). Symbols: cellobiose (□), glucose (●), dry cell mass (○), cellodextrin (■), and ethanol (▲). All values of fermentations are mean values from two independent fermentations, and error bars represent standard deviations.

Consequently, cellobiose fermentations with two *S. cerevisiae* strains expressing only GH1-1, BY-B and *TLG2*-knockout BY-B, were performed to validate whether deletion of *TLG2* gene could affect secretion of GH1-1 and accumulation of cellodextrin. Figs. 2A and 2B compare the cellobiose fermentation profiles between BY-B and BY-B (Δ*TLG2*) in YPC (20 g/l cellobiose, pH 6.5). Although BY-B showed almost the same fermentation profiles as previous fermentation under the same conditions (Fig. 2A), BY-B (Δ*TLG2*) showed considerably slower cell growth and cellobiose consumption with reduced ethanol production and decreased cellodextrin accumulation than BY-B (Fig. 2B). In cellobiose fermentation with BY-B (Δ*TLG2*), 2.3 g/l of maximum ethanol production and 3.9 g/l of maximum cellodextrin accumulation were observed, which corresponded to 45% and 37% reduction, respectively, compared with those observed in cellobiose fermentation with BY-B. Probably, *TLG2* knockout may hinder the non-conventional secretion of GH1-1, which could lead to delayed cellobiose metabolism in BY-B (Δ*TLG2*). As presented in Table 4, extracellular β-glucosidase activity assay performed on culture broth at the end of fermentations showed that secretion of GH1-1 from BY-B (Δ*TLG2*) decreased by 56% than that from BY-B [10.8 U/L in BY-B (Δ*TLG2*) vs. 24.5 U/L in BY-B], suggesting that *TLG2* knockout may prevent extracellular cellodextrin formation. However, it could not be excluded that *TLG2* knockout may inhibit cell growth, irrespective of preventing GH1-1 secretion.

To determine whether *TLG2* knockout could inhibit cell growth even under normal condition, glucose fermentations with BY-B and BY-B (Δ*TLG2*) were performed in YPD (20 g/l glucose, pH 6.5). As illustrated in Figs. 2C and 2D, the two strains showed almost the same fermentation profiles, including glucose consumption, cell growth and ethanol production, indicating that *TLG2* knockout did not affect cell growth and sugar metabolism at all. Interestingly, secretion of GH1-1 rarely occurred in both strains cultured in glucose [0.3 U/L in BY-B; 0.1 U/L in BY-B (Δ*TLG2*)] as shown in Table 4, which suggests that secretion of GH1-1 may be induced when there was a specific selective pressure, such as utilizing cellobiose as the sole carbon source, on cell growth. The results from cellobiose (or glucose) fermentations with BY-B and BY-B (Δ*TLG2*) are summarized in Table 5.

**Fig. 3:**
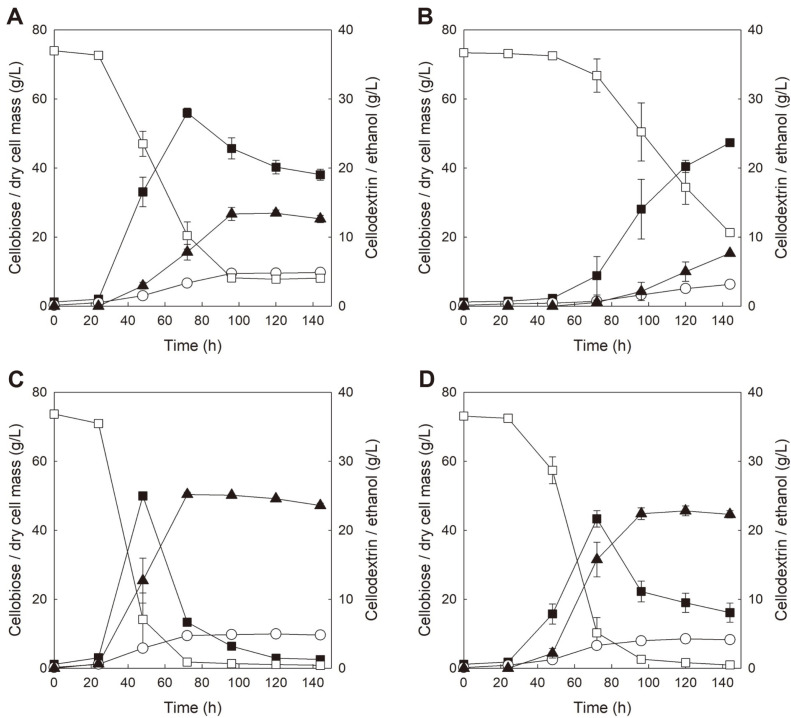
Comparison of fermentation profiles of *S. cerevisiae* strains expressing only GH1-1 [BY-B and BY-B (Δ*TLG2*)] and *S. cerevisiae* strains expressing both GH1-1 with CDT-1 [BY-BT and BY-BT (Δ*TLG2*)] in YP medium with 75 g/l cellobiose at pH 6.5. BY-B (**A**), BY-B (Δ*TLG2*) (**B**), BY-BT (**C**), and BY-BT (Δ*TLG2*) (**D**). Symbols: cellobiose (□), dry cell mass (○), cellodextrin (■), and ethanol (▲). All values of fermentations are mean values from two independent fermentations, and error bars represent standard deviations.

To elucidate whether similar patterns of cellobiose fermentation profiles could be observed when the concentration of cellobiose was increased, cellobiose fermentations with two strains were performed in YP containing 75 g/l cellobiose at pH 6.5. As shown in Figs. 3A and 3B, cellobiose fermentation profiles of BY-B and BY-B (Δ*TLG2*) under high cellobiose condition were similar to those under low cellobiose condition, respectively. During cellobiose fermentation with BY-B as shown in Fig. 3A, 65.9 g/l of cellobiose was consumed and 13.5 g/l of ethanol was produced along with accumulation and re-assimilation of cellodextrin from 28.0 g/l to 19.0 g/l. It is interesting to note that 8.1 g/l of cellobiose and 19.0 g/l of cellodextrin remained as residual sugars until the end of the fermentation, which was different from the result observed in fermentation with low concentration of cellobiose. Probably, the amount of GH1-1 secreted from BY-B is thought to be insufficient to degrade all of the cellobiose existing at high concentration. Furthermore, the total amount of GH1-1 that a single cell could secrete is thought to be maintained at a constant level, regardless of the initial cellobiose concentration. As shown in Table 4, specific extracellular β-glucosidase activity of BY-B grown in YPC75 was similar to BY-B grown in YPC20 (3.1 U/g cell in YPC75 vs. 2.9 U/g cell in YPC20 and 3.1 U/g cell in previous YPC20). Meanwhile, during cellobiose fermentation with BY-B (Δ*TLG2*) as shown in Fig. 3B, 52.1 g/l of cellobiose was consumed for production of 7.6 g/l ethanol and accumulation of 23.7 g/l cellodextrin, which decreased by 21%, 45%, and 15%, respectively, compared with cellobiose fermentation with BY-B. As mentioned above, it was supposed that *TLG2* knockout may limit secretion of GH1-1 and result in delayed cellobiose fermentation (with delayed cellodextrin formation). Table 4 indicates that extracellular secretion of GH1-1 from BY-B (Δ*TLG2*) grown in YPC75 was reduced by 57% than that from BY-B [13.0 U/L in BY-B (Δ*TLG2*) vs. 30.1 U/L in BY-B], which is similar to the result from fermentation in YPC20.

Because *TLG2* knockout was effective in preventing GH-1 secretion in cellobiose fermentation with BY-B (Δ*TLG2*), fermentations of 75 g/l cellobiose with BY-BT and BY-BT (Δ*TLG2*) were also tested to check whether *TLG2* knockout could affect cellodextrin accumulation and ethanol production in cellobiose fermentation with yeast strain expressing GH1-1 and CDT-1 together. As confirmed in Figs. 2B and 2D, the deletion of *TLG2* did not cause any phenotypic changes under YPD conditions but the deletion of *TLG2* prevented cellodextrin accumulation of BY-B (Δ*TLG2*) strain under YPC conditions. Taken together, we expected that *TLG2* deletion might affect cellodextrin accumulation and overall fermentation performances in YPC fermentation by BY-BT (Δ*TLG2*).

In cellobiose fermentation with BY-BT in YPC75 (pH 6.5) as shown in Fig. 3C, most of the initial cellobiose was consumed and 25.2 g/l of ethanol was produced at 72 h of the fermentation. Cellodextrin was accumulated to 25.0 g/l at 48 h and rapidly decreased until 72 h of fermentation, after which it decreased slowly. In particular, during the period for slow consumption of cellodextrin (72 h to 144 h), no more ethanol was produced, which may be the reason that cellodextrin accumulation reduced ethanol yield and productivity in cellobiose fermentation. In addition, secretion of GH1-1 from BY-BT was observed (38.6 U/L and 4.0 U/g cell) as shown in Table 4, which supports the result that cellodextrin accumulation by *S. cerevisiae* expressing GH1-1 and CDT could be due to the secretion of GH1-1. Fig. 3D shows cellobiose fermentation profiles of BY-BT (Δ*TLG2*) in YPC75 (pH 6.5). Unfortunately, considerably reduced cellobiose consumption, less production of ethanol, and slightly reduced cellodextrin accumulation were observed in BY-BT (Δ*TLG2*), although secretion of GH1-1 decreased by more than 40% compared with BY-BT [21.8 U/L in BY-BT (Δ*TLG2*) vs. 38.6 U/L in BY-BT] as shown in Table 4. *TLG2* knockout led to retardation of cellodextrin formation; however, cellobiose consumption and ethanol production were also delayed. In particular, the accumulated cellodextrin was re-assimilated considerably slowly, and 8.1 g/l of cellodextrin remained until the end of the fermentation. These results suggest that *TLG2* knockout may affect the function (or expression or delivery) of CDT-1 because rapid consumption of cellodextrin as well as cellobiose was observed in the case of cellobiose fermentation with BY-BT. Consequently, *TLG2* knockout is assumed to have negative effects on cellobiose metabolism in *S. cerevisiae* expressing GH1-1 and CDT-1, although it could prevent non-conventional secretion of GH1-1. The results from cellobiose fermentations with four strains [BY-B, BY-B (Δ*TLG2*), BY-BY, and BY-B (Δ*TLG2*)] are summarized in Table 5.

Cellodextrin accumulation by *S. cerevisiae* expressing GH1-1 and CDT has been regarded as one of the main problems decreasing fermentation performance in terms of ethanol yield and productivity in the cellobiose fermentation [16-18]. In this study, it was observed that secretion of a small amount of GH1-1 could cause extracellular formation of cellodextrin. Furthermore, it has been observed that knockout of *TLG2*, involved in non-conventional protein secretion, was effective in reducing the secretion of GH1-1 lacking signal peptide for classical protein secretion, resulting in delayed and reduced cellodextrin accumulation in cellobiose fermentation with *S. cerevisiae* expressing only GH1-1. However, *TLG2* knockout negatively influenced on cellodextrin accumulation in cellobiose fermentation with *S. cerevisiae* expressing both GH1-1 and CDT-1, resulting in delayed cellobiose consumption and no significant change in cellodextrin accumulation. Perhaps, other genes involved in non-conventional protein secretion [24, 26] need to be investigated for prevention of GH1-1 secretion and reduction of cellodextrin formation in *S. cerevisiae* expressing GH1-1 and CDT-1. Probably, additional knockout of other genes along with *TLG2* might be effective on cellodextrin accumulation in *S. cerevisiae* fermenting cellobiose. In addition, unknown properties of heterologous GH1-1 may stimulate its secretion from *S. cerevisiae* because GH1-1 was originated from fungi [11], which suggests that other intracellular β-glucosidase genes from other yeast species (*e.g.*, *BGL* genes from *Pichia stipitis*) [30] need to be tested to confirm whether they are also secreted through non-conventional protein secretion in *S. cerevisiae*.

## Figures and Tables

**Table 1:** The list of plasmids and *S. cerevisiae* strains used in this study.

Plasmids and strains	Relevant features	Sources
Plasmids		
pRS425PGK	*LEU2*, P_PGK_-MCS-T_CYC_, 2μ origin, Amp^r^	[11]
pRS426PGK	*URA3*, P_PGK_-MCS-T_CYC_, 2μ origin, Amp^r^	[11]
pRS425-gh1-1	*LEU2*, P_PGK_-*gh1-1*-T_CYC_, 2μ origin, Amp^r^	[11]
pRS426-cdt1		
Strains	*URA3*, P_PGK_-*cdt1*-T_CYC_, 2μ origin, Amp^r^	[11]
BY4742	*MATα*, *his3*, *leu2*, *lys2*, *ura3*	This study
BY4742 (Δ*TLG2*)	BY4742, *tlg2Δ::KanMX*	This study
BY-B	BY4742/pRS425-gh1-1/pRS426PGK	This study
BY-B (Δ*TLG2*)	BY4742 (Δ*TLG2*)/pRS425-gh1-1/pRS426PGK	This study
BY-BT	BY4742/pRS425-gh1-1/pRS426-cdt1	This study
BY-BT (Δ*TLG2*)	BY4742 (Δ*TLG2*)/pRS425-gh1-1/pRS426-cdt1	This study

**Table 2:** Extracellular β-glucosidase accumulation by engineered *S. cerevisiae* expressing GH1-1 (BY-B) under different culture conditions.

Culture conditions	Volumetric extracellular β-glucosidase activity in culture broth (U/L)	Specific extracellular β-glucosidase activity (U/g cell)
YPC (20 g/l cellobiose, pH 6.5)	14.9 ± 3.80	3.1 ± 0.80
YPC (20 g/l cellobiose, pH 5.5)	14.5 ± 2.96	3.1 ± 0.64
SCC (20 g/l cellobiose, pH 6.5)	0.6 ± 0.32	0.7 ± 0.34
SCC (20 g/l cellobiose, pH 5.5)	0.0	0.0

The culture broth at the end (72 h) of the fermentation periods were used for the determination of extracellular β-glucosidase activity. All values are mean values from the samples of two independent fermentations, and error bars represent standard deviations.

**Table 3:** Summarized results from micro-aerobic fermentations of engineered *S. cerevisiae* strain expressing GH1-1 (BY-B) under different culture conditions.

Culture conditions	Final dry cell mass (g/l)	Final sugar (g/l)	Maximum ethanol (g/l)	Maximum cellodextrin (g/l)
YPC (20 g/l cellobiose, pH 6.5)	4.8 ± 0.13	2.6 ± 0.41	4.0 ± 0.04	7.2 ± 0.88
YPC (20 g/l cellobiose, pH 5.5)	4.6 ± 0.45	2.2 ± 0.30	4.6 ± 0.75	7.7 ± 0.75
SCC (20 g/l cellobiose, pH 6.5)	0.9 ± 0.11	14.8 ± 1.74	0.0	3.4 ± 0.66
SCC (20 g/l cellobiose, pH 5.5)	0.1 ± 0.01	20.3 ± 0.05	0.0	0.0

Fermentation experiments were performed for 72 h. All values are mean values from samples of two independent fermentations, and error bars represent standard deviations.

**Table 4:** Extracellular β-glucosidase accumulation by engineered *S. cerevisiae* strains in YP medium with cellobiose (or glucose) at pH 6.5.

Culture conditions	Strains	Volumetric extracellular β-glucosidase activity in culture broth (U/L)	Specific extracellular β-glucosidase activity (U/g cell)
YPC20	BY-B	24.5 ± 2.76	2.9 ± 0.33
	BY-B (Δ*TLG2*)	10.8 ± 0.90	2.1 ± 0.17
YPD20	BY-B	0.3 ± 0.15	0.1 ± 0.04
	BY-B (Δ*TLG2*)	0.1 ± 0.11	0.0 ± 0.03
YPC75	BY-B	30.1 ± 2.61	3.1 ± 0.27
	BY-B (Δ*TLG2*)	13.0 ± 1.80	2.1 ± 0.29
	BY-BT	38. 6 ± 7.41	4.0 ± 0.77
	BY-BT (Δ*TLG2*)	21.8 ± 2.39	2.6 ± 0.29

The culture broth at the end (120, 36, and 144 h for YPC20, YPD20, and YPC75, respectively) of the fermentation periods were used for determination of extracellular β-glucosidase activity. All values are mean values from samples of two independent fermentations, and error bars represent standard deviations.

**Table 5:** Summarized results from micro-aerobic fermentations of engineered *S. cerevisiae* strains in YP medium with cellobiose (or glucose) at pH 6.5.

Culture conditions	Strains	Final dry cell mass (g/l)	Final sugar (g/l)	Maximum ethanol (g/l)	Maximum cellodextrin (g/l)
YPC20	BY-B	8.4 ± 0.69	0.3 ± 0.03	4.2 ± 0.68	6.2 ± 0.48
	BY-B (Δ*TLG2*)	5.1 ± 0.07	5.8 ± 0.08	2.3 ± 0.03	3.9 ± 0.43
YPD20	BY-B	3.9 ± 0.14	0.0	7.8 ± 0.01	0.0
	BY-B (Δ*TLG2*)	3.6 ± 0.21	0.0	7.9 ± 0.10	0.0
YPC75	BY-B	9.7 ± 0.17	8.1 ± 0.58	13.5 ± 0.46	28.0 ± 0.68
	BY-B (Δ*TLG2*)	6.3 ± 0.06	21.3 ± 0.03	7.6 ± 0.06	23.7 ± 0.56
	BY-BT	9.6 ± 0.09	0.9 ± 0.05	25.2 ± 0.33	25.0 ± 0.26
	BY-BT (Δ*TLG2*)	8.3 ± 0.69	1.0 ± 0.28	22.8 ± 0.72	21.7 ± 1.19

Fermentation experiments with YPC20, YPD20, and YPC75 were performed for 120, 36, and 144 h, respectively.
